# Supplementary material for: Spontaneous current constriction in threshold switching devices
Source: Nat Commun. 2019 Apr 9;10:1628. doi: 10.1038/s41467-019-09679-9 (PMC6456614; doi:10.1038/s41467-019-09679-9)
Supplement: Supplementary file 1 — Supplementary Information [file 41467_2019_9679_MOESM1_ESM.pdf]

Spontaneous current constriction in threshold switching devices

Goodwill et al.

Supplementary Information

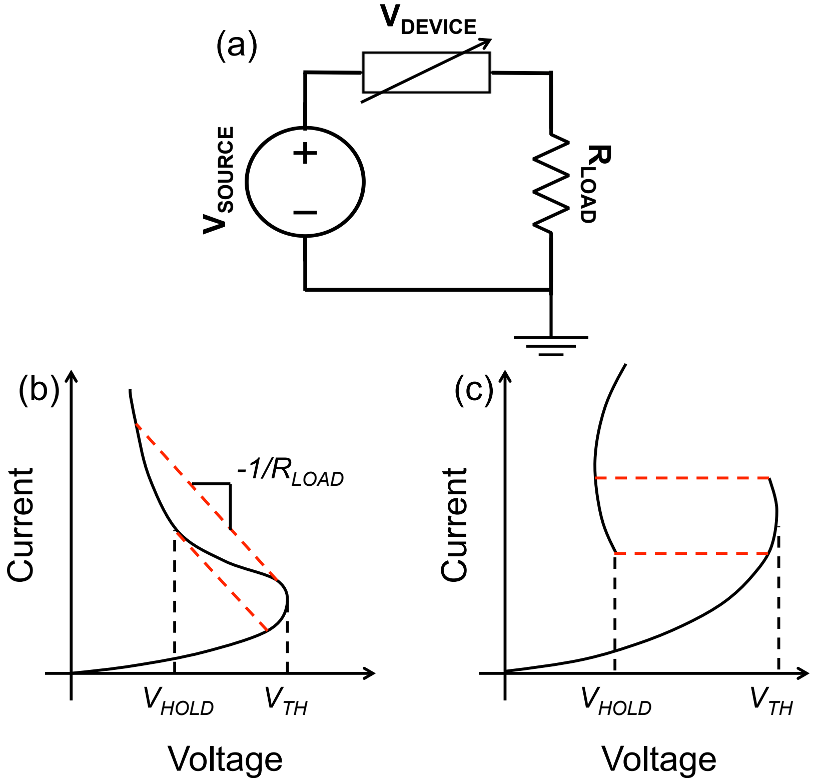

Supplementary Figure 1. Schematic of testing set-up and observed TaO_x *I-V* characteristics. (a) Electrical testing circuit layout for *I-V* characterization and SJEM. Schematic of (b) S-NDR *I-V* and (c) multivalued *I-V*.

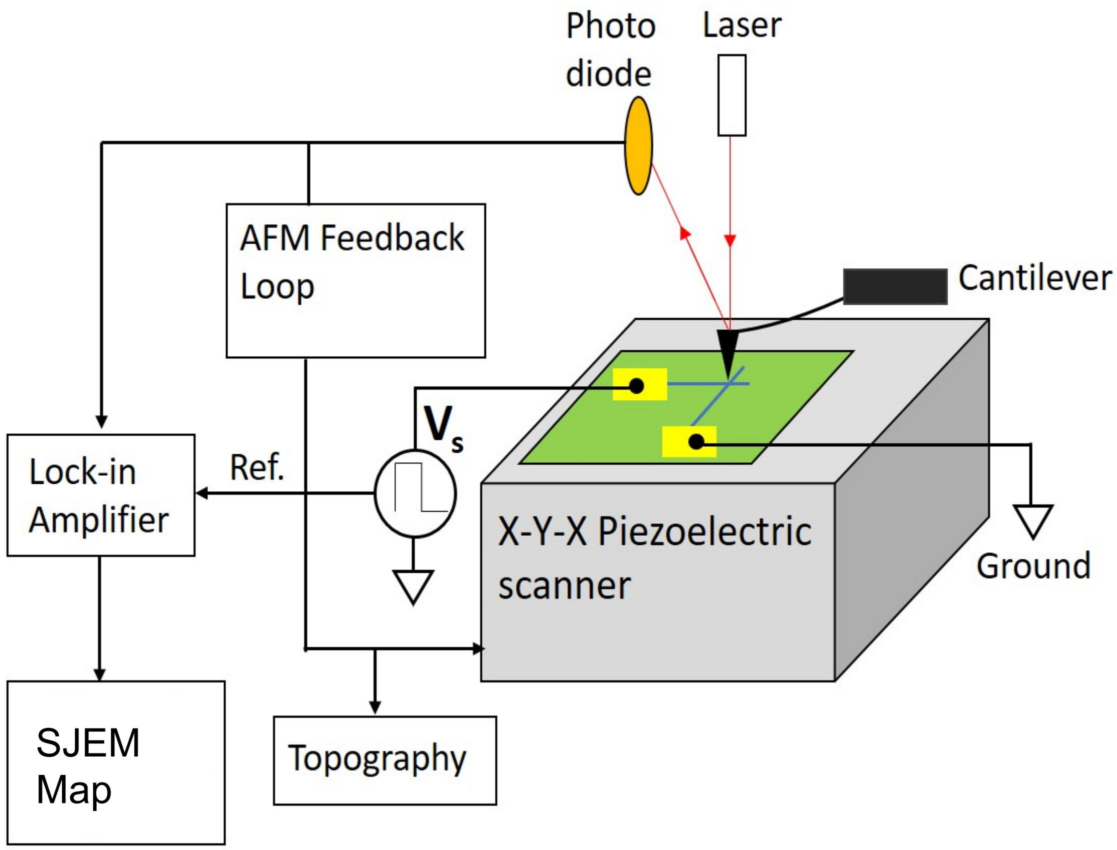

Supplementary Figure 2. Schematic of SJEM measurement setup.

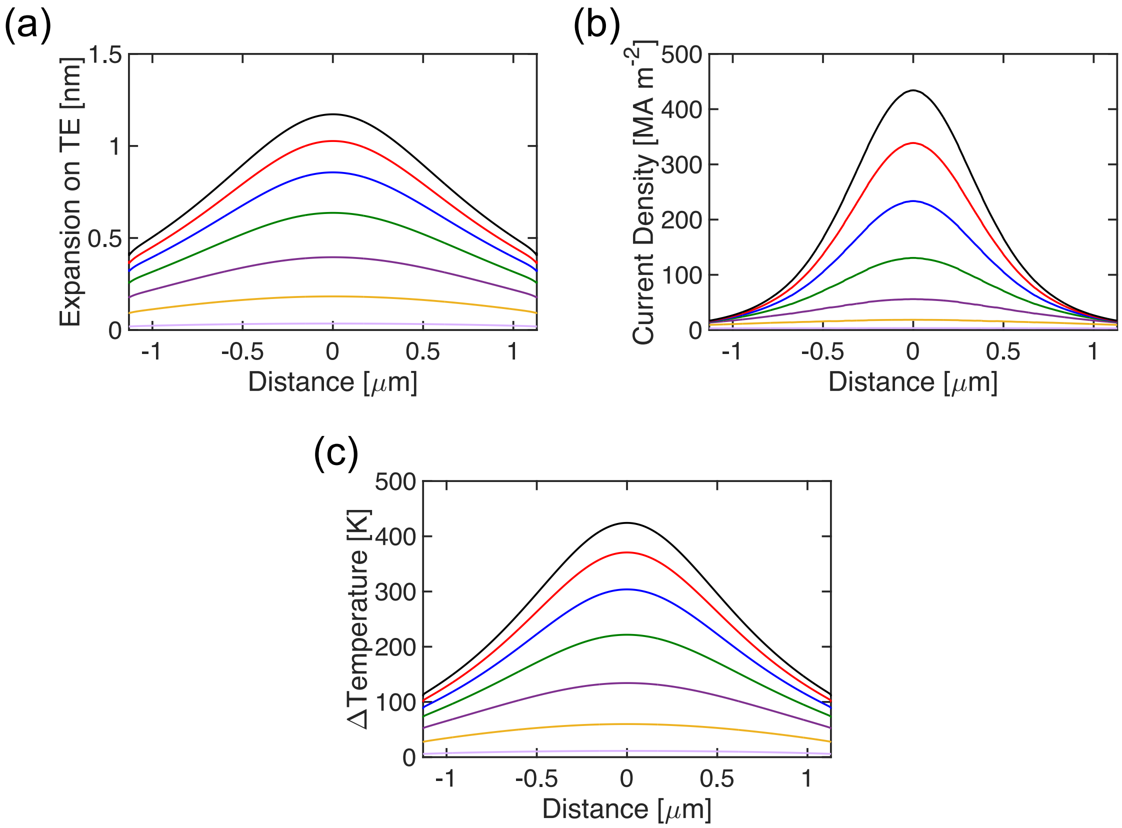

Supplementary Figure 3. Simulated characteristics of a 2 × 2 μm TaO_x device. Simulated line profiles of (a) expansion (b) current density and (c) temperature for all points of equal power dissipation along the *I-V* shown in Figure 2(b). Source data are provided as a Source Data file.

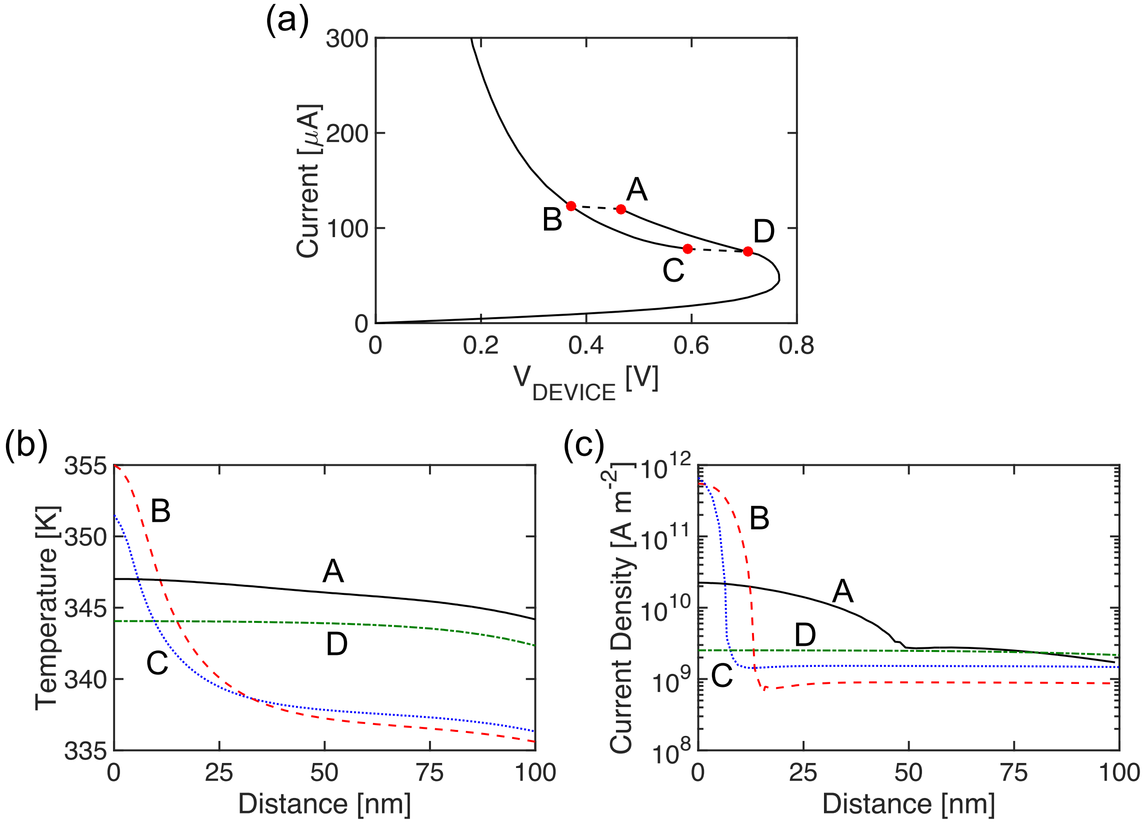

Supplementary Figure 4. Simulated characteristics of a 200 nm diameter VO_2 device. (a) Simulated *I-V* for VO_2-based device with current source, shown for reference from Figure 5. (b) Temperature and (c) current density line profiles in the device shown for points A-D along the *I-V* curve. Source data are provided as a Source Data file.

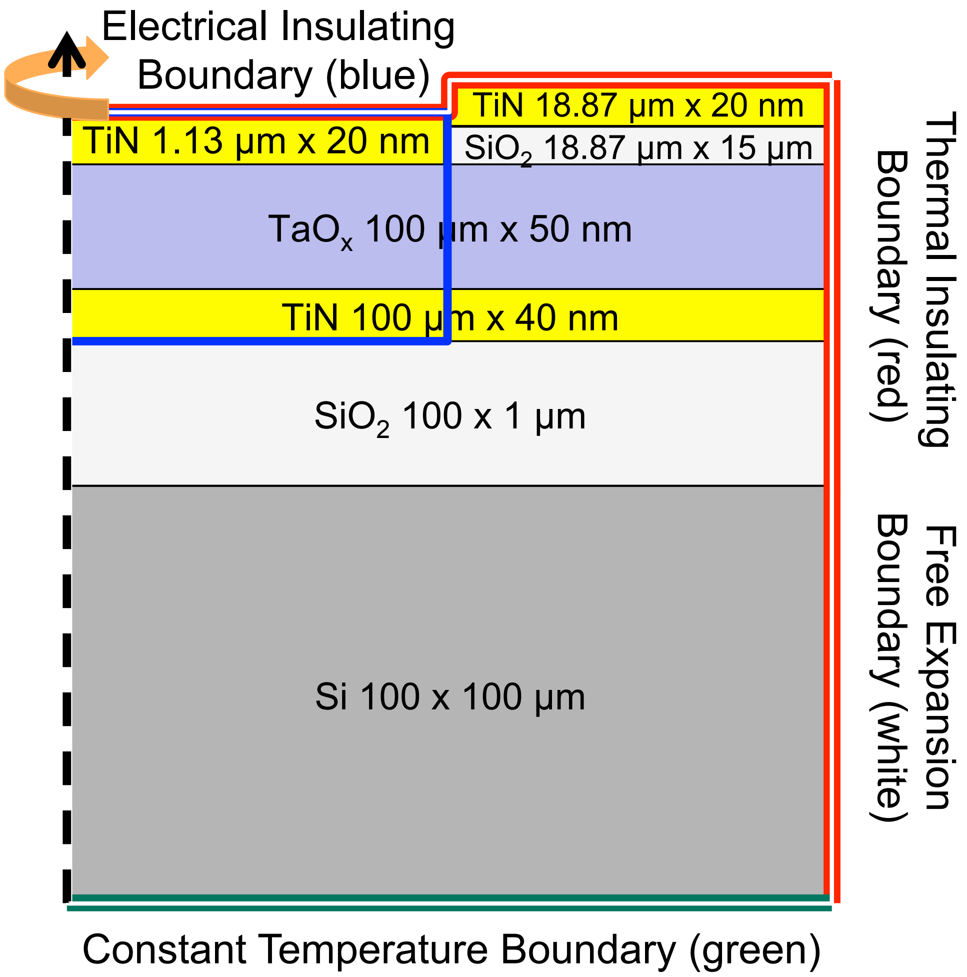

Supplementary Figure 5. Device structure and boundary conditions used in simulation. The dimensions shown for each layer are indicated as width × height.

Supplementary Table 1. Material properties used in simulation.

	Si	SiO_2	TiN[1]	TaO_x[1]
Density ($kg \cdot m^{-3}$)	2329	2200	5210	8200
Thermal conductivity ($W \cdot m^{-1} \cdot K^{-1}$)	130	1.4	5	4
Electrical conductivity ($S \cdot m^{-1}$)	N/A	N/A	5×10^{6}	User defined
Heat capacity ($J \cdot kg^{-1} \cdot K^{-1}$)	700	730	545	174
Relative permittivity	N/A	N/A	4	22
Thermal expansion coefficient (1/K)	2.6×10^{-6}	0.5×10^{-6}	1.0×10^{-5} [2]	5.0×10^{-6} [3]
Young's Modulus (GPa)	170	70	300[2]	179[3]
Poisson's Ratio	0.28	0.17	0.25[2]	0.27[3]

Supplementary References

1. Goodwill, J. M., Sharma, A. A., Li, D., Bain, J. A. & Skowronski, M. Electro-Thermal Model of Threshold Switching in TaOx-Based Devices. *ACS Appl. Mater. Interfaces* **9,** 11704–11710 (2017).

2. Cen, Z. H. *et al.* Temperature effect on titanium nitride nanometer thin film in air. *J. Phys. D. Appl. Phys.* **50,** (2017).

3. Braginsky, V. B. & Samoilenko, A. A. Measurements of the optical mirror coating properties. *Phys. Lett. A* **315,** 175–177 (2003).
